# Palliative care for patients with HIV/AIDS admitted to intensive care units

**DOI:** 10.5935/0103-507X.20160054

**Published:** 2016

**Authors:** Paola Nóbrega Souza, Erique José Peixoto de Miranda, Ronaldo Cruz, Daniel Neves Forte

**Affiliations:** 1 Instituto de Infectologia Emílio Ribas - São Paulo (SP), Brazil.; 2 Hospital Universitário, Universidade de São Paulo - São Paulo (SP), Brazil.; 3 Intensive Care Team, Hospital Sírio-Libanês - São Paulo (SP), Brazil.

**Keywords:** Palliative care, HIV, Acquired immunodeficiency syndrome, Intensive care units

## Abstract

**Objective:**

To describe the characteristics of patients with HIV/AIDS and to compare the therapeutic interventions and end-of-life care before and after evaluation by the palliative care team.

**Methods:**

This retrospective cohort study included all patients with HIV/AIDS admitted to the intensive care unit of the *Instituto de Infectologia Emílio Ribas* who were evaluated by a palliative care team between January 2006 and December 2012.

**Results:**

Of the 109 patients evaluated, 89% acquired opportunistic infections, 70% had CD4 counts lower than 100 cells/mm^3^, and only 19% adhered to treatment. The overall mortality rate was 88%. Among patients predicted with a terminally ill (68%), the use of highly active antiretroviral therapy decreased from 50.0% to 23.1% (p = 0.02), the use of antibiotics decreased from 100% to 63.6% (p < 0.001), the use of vasoactive drugs decreased from 62.1% to 37.8% (p = 0.009), the use of renal replacement therapy decreased from 34.8% to 23.0% (p < 0.0001), and the number of blood product transfusions decreased from 74.2% to 19.7% (p < 0.0001). Meetings with the family were held in 48 cases, and 23% of the terminally ill patients were discharged from the intensive care unit.

**Conclusion:**

Palliative care was required in patients with severe illnesses and high mortality. The number of potentially inappropriate interventions in terminally ill patients monitored by the palliative care team significantly decreased, and 26% of the patients were discharged from the intensive care unit.

## Abstract (pt)

**Objetivo:**

Descrever as características de pacientes com HIV/AIDS e comparar as medidas terapêuticas e cuidados de fim de vida, antes e após a avaliação da equipe de cuidados paliativos.

**Métodos:**

Trata-se de uma coorte retrospectiva, que incluiu todos os pacientes com HIV/AIDS internados na unidade de terapia intensiva do Instituto de Infectologia Emílio Ribas e avaliados pela equipe de cuidados paliativos no período de janeiro de 2006 a dezembro de 2012.

**Resultados:**

Dos 109 pacientes avaliados, 89% tinham relato de doenças oportunistas, 70% apresentavam CD4 menor que 100cels/mm^3^ e apenas 19% aderiram ao tratamento. A mortalidade geral foi de 88%. Dentre os pacientes considerados em provável terminalidade (68%), observaram-se redução do uso da terapia antirretroviral altamente ativa (50,0% para 23,1%; p = 0,02), antibioticoterapia (100% para 63,6%; p < 0,001), drogas vasoativas (62,1% para 37,8%, p = 0,009), terapia de substituição renal (34,8% para 23%; p < 0,0001) e transfusão de hemoderivados (74,2% para 19,7%; p < 0,0001). Foi realizada reunião com a família em 48% dos casos, e 23% dos pacientes em provável terminalidade receberam alta da unidade de terapia intensiva.

**Conclusão:**

A equipe de cuidados paliativos foi acionada para pacientes com perfil de elevadas gravidade e mortalidade. Os pacientes acompanhados pela equipe de cuidados paliativos avaliados como provável terminalidade tiveram significativa redução de intervenções potencialmente inapropriadas, e 26% desses pacientes conseguiram receber alta da unidade de terapia intensiva.

## INTRODUCTION

It is estimated that 36.9 million people live with HIV/AIDS worldwide, with approximately 2 million new cases and 1.2 million deaths per year.^(1)^ In Brazil, approximately 781,000 individuals live with HIV/AIDS, and 12,449 deaths were recorded in 2014.^(2)^

In the early years of the HIV epidemic, the hospital survival rate of patients admitted to the intensive care unit (ICU) was approximately 30% and was less than 15% for those who progressed to respiratory failure.^(3)^ After improvements in intensive care and the introduction of highly active antiretroviral therapy (HAART) in 1996, HIV became a chronic disease, and infected patients could live longer.^(3,4)^ Although survival in the ICU has improved since the beginning of the HIV epidemic, the mortality rate of critically ill patients is approximately 30%, which is still higher than the mortality rate in patients not infected with HIV.^(5,6)^

Mortality in the ICU is often accompanied by aggressive interventions, costly treatments, inadequate control of symptoms, and social isolation.^(7)^ In this context, palliative care is essential for decisions on the limitation of invasive procedures, management of symptoms, and end-of-life care.^(8,9)^

In 2002, the World Health Organization defined palliative care as a strategy aimed at reducing the suffering of patients with life-threatening illnesses.^(10)^ Since then, several medical societies that serve as references for intensive care began to incorporate palliative care as an integral part of the quality of care for critically ill patients. Therefore, the evaluation of the management of symptoms, the alignment of the treatments to the beliefs and preferences of the patients, and the recognition of the problem of dysthanasia began to be considered as quality interventions in the ICU.^(8,9,11-17)^ It was recognized that palliative care should be used not only in the terminal phase but also to manage any serious illnesses; additionally, palliative care should be integrated with health care focused on the cure or management of the disease through its course and not only in the terminal phase,^(10)^ which is particularly important in the ICU setting.^(18)^

In this context, primary palliative care should be adopted by all health professionals who deal with severe illnesses. This strategy can be achieved by training these professionals^(19)^ and acknowledging that the indication for palliative care should be based on the needs and not on the diagnosis and prognosis of the patient.^(10)^ Therefore, the evaluation of specialists would be appropriate in selected cases according to the characteristics and needs of the institution in question. This consultative model can promote the practice of palliative care in the ICU and improve the quality of care for patients and their families.^(20)^

In Brazil, few studies have evaluated the initiatives for the integration of palliative care with intensive care. This study aimed to describe the characteristics of patients with HIV/AIDS admitted to an intensive care unit who were evaluated by a palliative care team and to compare the therapeutic interventions and end-of-life care before and after the evaluation of terminally ill patients.

## METHODS

The study protocol was reviewed and approved by the Scientific Committee of the *Instituto de Infectologia Emílio Ribas* (IIER) and the Research Ethics Committee of the *Hospital Sírio-Libanês* under protocol Nos. 42/2014 and 53/2014, respectively.

This retrospective cohort study was conducted in the IIER between January 2006 and December 2012. The IIER is a public tertiary university hospital and a reference for infectious diseases in the state of São Paulo. At the time of the study, the hospital had 199 beds, 17 of which were in the ICU. The palliative care team of the hospital has provided consultation since 1999 upon request by the treating physician. The team was composed of two doctors, two nurses, one nursing assistant, two social workers, one occupational therapist, one nutritionist, and three chaplains.

The inclusion criteria were patients admitted to the ICU of IIER during the study period with a confirmed diagnosis of HIV/AIDS who requested a consultation in the Interdisciplinary Center for Palliative Care (*Núcleo Interdisciplinar de Cuidados Paliativos* - NICP). Potentially eligible patients were selected from the official registry of the NICP and the record of initial evaluation used by the palliative care team (*Fichas de Avaliação Inicial da Equipe de Cuidado Paliativo* - FAICP). Both documents were kept in the NICP. The official registry contained all consultation requests for the palliative care team. The FAICP contained underlying diseases, HIV/AIDS status (CD4 count, current and previous opportunistic diseases, and level of adherence to treatment), reason for hospitalization in the ICU and requests for a palliative care consultation. After analyzing the registry book and FAICP, the respective hospital records were requested from the Medical Records and Statistics Service (*Serviço de Atendimento Médico e Estatístico* - SAME). In cases in which there was a divergence of information between the FAICP and the hospital records, the data present in the hospital records prevailed. To better evaluate the study sample, the number of patients hospitalized in the ICU during the study period, the frequency of patients diagnosed with HIV/AIDS, and the overall mortality in the ICU were also calculated.

Patients with requests for a palliative care consultation who died before the evaluation were excluded from the analysis. Two main analyses were conducted. The first involved all patients with requests for a palliative care consultation. In this first analysis, we described the demographic characteristics of patients, CD4 counts, opportunistic diseases, time since diagnosis, adherence to treatment, and reason for and length of stay in the ICU. This analysis was based on the data from the FAICP and hospital records.

In the second analysis, the therapeutic interventions and end-of-life care before and after the evaluation of the terminally ill patients were compared using only data from patients with available records because data related to changes in the therapeutic interventions were present only in the hospital records.

The statistical analysis was conducted using the Statistical Package for Social Sciences (SPSS) software version 20.0 for Windows. The variables were subjected to a normality analysis using the Kolmogorov-Smirnov test. The normally distributed numerical variables are expressed as the mean and standard deviation, and the groups were compared using Student's *t*-test and analysis of variance (ANOVA). The numerical variables with non-normal distributions are expressed as the median and interquartile range (IQR) and compared using the Mann-Whitney test and Kruskal-Wallis test. The categorical variables are expressed as absolute frequencies (N) and relative frequencies (percentages) and compared using Fisher's exact test and the Chi-square test (for unpaired data) or the McNemar test (for paired data). The graphs were prepared using GraphPad Prism version 6.0. A Kaplan-Meier curve was graphed with the log-rank test to compare survival rates between the groups of terminally ill patients, non-terminally ill patients, and patients with an indeterminate prognosis. P-values smaller than 0.05 were considered statistically significant.

## RESULTS

**Figure 1:**
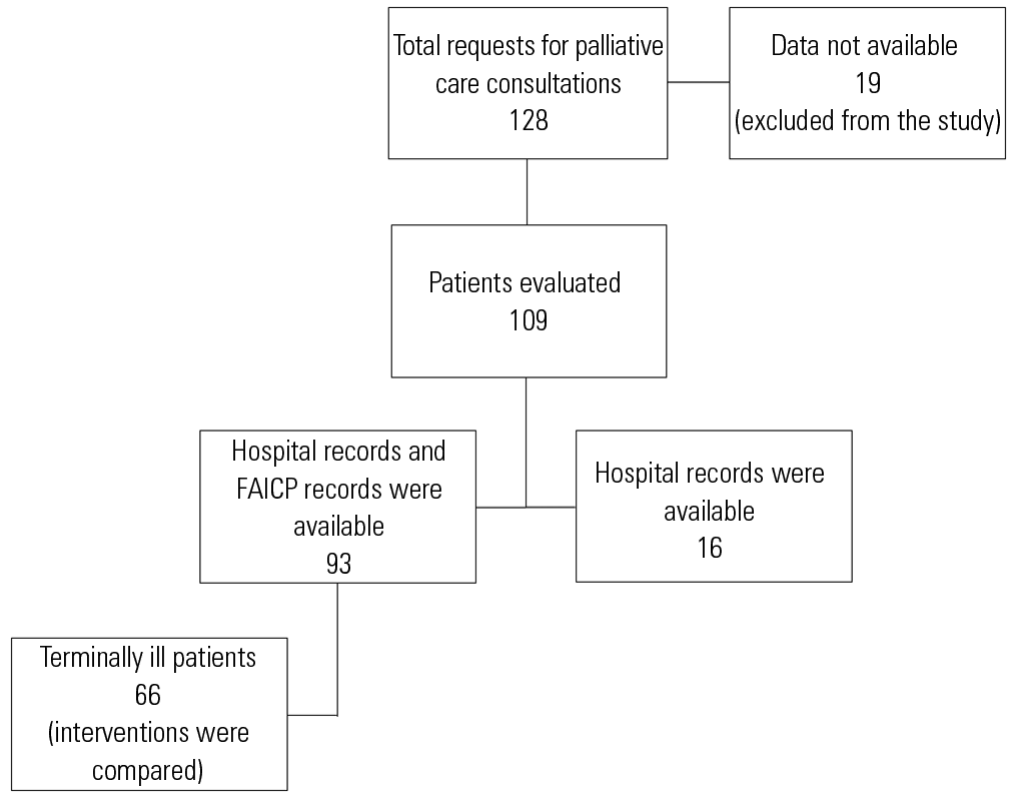
Organization chart of patients included in the study and evaluation of FACIP records and hospital records. FAICP - *Fichas de Avaliação Inicial da Equipe de Cuidado Paliativo.*

Of the 128 patients with requests for a palliative care consultation, 19 were excluded from the analysis because they died before the evaluation by the palliative care team; therefore, 109 patients were included in the descriptive analysis (Figure 1). During the study period, there were 3,738 ICU admissions, 2,233 patients were diagnosed with HIV/AIDS (60% of the total admissions), and the hospital mortality rate was 31%. Additionally, 6% (128/2,233) of the HIV/AIDS patients admitted to the ICU had requests for a palliative care consultation, and 85% (109/128) of these consultations were performed.

The primary reasons for ICU admission of the evaluated patients were acute respiratory failure (50/109; 46%), followed by a decreased level of consciousness, sepsis, and renal failure. In 71% (77/109) of the cases, the HIV/AIDS-related diseases were responsible for the ICU admission (Table 1). Previous opportunistic infections were diagnosed in 51% (56/109) of the cases, and active opportunistic infections were diagnosed in 82% (89/109) of the cases, with a predominance of tuberculosis (47%; 51/109).

**Table 1:** Characteristics of the patients with HIV/AIDS admitted to the intensive care unit with requests for palliative care consultations

Characteristics	
Male (N = 109) (%)	74 (67.9)
Age (years) (N = 109)*	40.57 ± 10.89 (11 - 75)
Days of hospitalization prior to admission to the ICU (N = 99)†‡	9 (2 - 24), 0 -110
Days of hospitalization in the ICU until request for palliative care consultation (N = 99)†‡	9 (2 - 22.5), 0 - 124
Length of ICU stay (days) (N = 99)†‡	21 (8.5 - 39.0), 0 - 178
Length of hospital stay (days) (N = 108)†‡	45.5 (24 - 60), 1 - 217
Duration of palliative care (days) (N = 109)†	7 (2 - 19), 0 - 75
Reason for ICU admission (N = 109)§	
Acute respiratory failure	50 (46)
Decreased level of consciousness	35 (32)
Sepsis and/or shock	19 (17)
Renal failure	18 (17)
Postoperative complications	5 (5)
Other	5 (5)
More than one of the above	26 (24)
Unknown	5 (5)
Admission to the ICU due to HIV/AIDS-related diseases (N = 109)	
HIV/AIDS-related	76 (70)
Not HIV/AIDS-related	28 (26)
Unknown	5 (5)
Opportunistic infections (N = 109)§	
Total	94 (86)
Previous	56 (51)
Current	89 (82)
None	12 (11)
Unknown	3 (3)
Types of opportunistic infections diagnosed (N = 109)§	
Tuberculosis	51 (47)
Brain toxoplasmosis	28 (26)
Cytomegalovirus	18 (17)
Neurocryptococcosis	14 (13)
Pneumocystis pneumonia	12 (11)
Kaposi's sarcoma	10 (9)
Progressive multifocal leukoencephalopathy	6 (6)
Mycobacteriosis due to *Mycobacterium avium-intracellulare*	6 (6)
Lymphoma	4 (4)
Esophageal candidiasis	3 (3)
Cryptosporidiosis	2 (2)
Histoplasmosis	2 (2)
Other	10 (9)
More than one of the above	53 (49)

ICU - intensive care unit.

* Data with a normal distribution, expressed as the mean ± standard deviation and variation (minimum-maximum);

† data without a normal distribution, expressed as the median and interquartile range (Q1-Q3) and variation (minimum-maximum);

‡ N was lower than the total due to data unavailability;

§ the sum is greater than 100% because some patients belonged to more than one category. Values are expressed as the mean ± standard deviation and number (%).

The median time since HIV diagnosis was eight years. The median CD4 count was 39 cells/mm^3^ and was lower than 100cells/mm^3^ in 70% (76/109) of the cases (Table 2).

**Table 2:** Characteristics of the patients with HIV/AIDS admitted to the intensive care unit with requests for palliative care consultations (N = 109)

Characteristics	Terminally ill	Non-terminally ill	Indeterminate prognosis	Total	p-value*
	**N (%)**	**N (%)**	**N (%)**	**N (%)**	
Period of diagnosis					
< 1 year	11 (15)	3 (20)	6 (30)	20 (18)	0.444
1 - 5 years	13 (18)	2 (13)	1 (5)	16 (15)	
> 5 years	45 (60)	10 (67)	11 (55)	66 (61)	
Vertical transmission	3 (4)	0	2 (10)	5 (5)	
Unknown†	2 (3)	0	0	2 (2)	
CD4 T-lymphocyte count					
< 100 cells/mm^3^	54 (73)	8 (53)	14 (70)	76 (70)	0.453
101 - 200 cells/mm^3^	11 (15)	5 (33)	3 (15)	19 (17)	
> 200cells/mm^3^	7 (9)	2 (13)	3 (15)	12 (11)	
Unknown†	2 (3)	0	0	2 (2)	
Adherence to treatment					
Yes	14 (19)	2 (13)	5 (25)	21 (19)	0.542
No	46 (62)	9 (60)	9 (45)	64 (59)	
Not applicable‡	11 (15)	3 (20)	5 (25)	19 (17)	
Unknown†	3 (4)	1 (7)	1 (5)	5 (5)	
Outcome					
Death	73 (99)	10 (67)	13 (65)	96 (88)	<0.0001
Hospital discharge	1 (1)	5 (33)	7 (35)	13 (17)	
Place of death					
ICU	49 (67)	6 (40)	8 (40)	63 (58)	
Ward	16 (22)	1 (7)	1 (5)	18 (17)	
Unknown‡§	8 (11)	4 (20)	4 (20)	15 (14)	
Total	74	15	20	109	

ICU - intensive care unit.

* McNemar test (for paired samples) or when indicated * Fisher's exact test or Chi-square test (for independent samples), both two-tailed tests;

† records unavailable or with incomplete data;

‡ included newly diagnosed cases in which patient adherence was not evaluated;

§ indeterminate place of death.

The data on the previous use of medications indicated that 59% (62/105) of the patients were using HAART and 24% (24/100) were under prophylaxis for opportunistic infections. However, only 19% (21/109) of the patients adhered to treatment, and 59% (64/109) of the patients reported irregular treatment or dropout.

The median hospital stay was 45.5 days, and the median ICU stay was 21 days. The median follow-up period by the palliative care team was seven days. Among the patients evaluated, 96 (88%) died and 13 (11.9%) were discharged.

**Figure 2:**
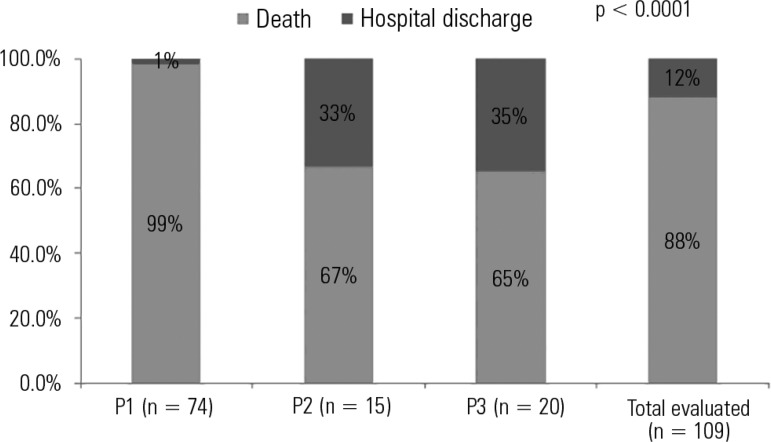
Relationship between the defined prognosis and mortality in patients with HIV/AIDS admitted to the intensive care unit and evaluated by the palliative care team (N = 109). P1 - terminally ill patients; P2 - non-terminally ill patients; P3 - indeterminate prognosis.

**Figure 3:**
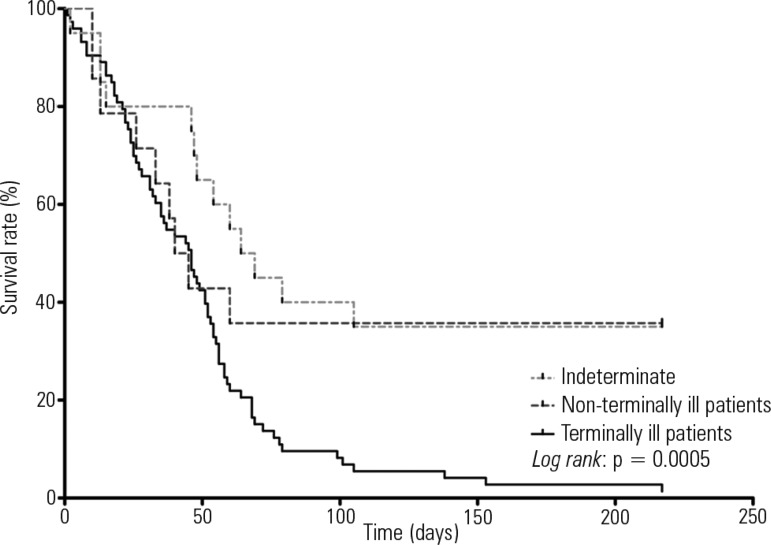
Kaplan-Meier curve and survival for patients with different prognoses.

In the initial evaluation by the palliative care team, 68% (74/109) of the patients were predicted with a terminal illness, 14% (15/109) of the patients were predicted with a non-terminal illness, and 18% (20/109) of the patients had an indeterminate prognosis. The hospital mortality rate was higher among the terminally ill patients than among the patients predicted with a non-terminal illness or with an indeterminate prognosis, corresponding to 99% (73/74), 65% (13/20), and 67% (10/15) (p < 0.0001), respectively (Figures 2 and 3).

Among the 74 patients evaluated by the palliative care team who were predictedwith a terminal illness, eight patients did not have records available. Therefore, the prescriptions before and after the evaluation were compared in 66 patients. Our results indicated that the prescription of HAART, prophylaxis for opportunistic infections, blood product transfusions, and the use of antibiotics decreased significantly after the consultation and prognosis (p < 0.05 for all of these variables) (Table 3). There was no significant difference in the use of antituberculosis drugs, sedation, and analgesia.

**Table 3:** Therapy administered before and after palliative care among terminally ill patients with HIV/AIDS (N = 66)

Therapy administered	Before palliative care	After palliative care	p-value*
	N (%)	N (%)	
HAART‡	33/66 (50)	15/66 (23)	0.02
Prophylaxis against opportunistic diseases	47/66 (71)	33/66 (50)	0.02
Antibiotics	66/66 (100)	42/66 (64)	< 0.001
Cycles of antibiotics			
1	9/66 (14)		
> 1	57/66 (86)		
Antituberculosis drugs	30/66 (45)	23/66 (35)	0.287
Sedation	50/66 (76)	40/66 (61)	0.092
Analgesia	60/66 (91)	48/66 (73)	0.236
Type of analgesia†			
Non-opioids	9/66 (14)	19/66 (29)	
Codeine/tramadol	5/66 (8)	4/66 (6)	
Morphine	13/66 (19)	16/66 (24)	
Methadone	3/66 (5)	2/66 (3)	
Fentanyl	51/66 (77)	41/66 (62)	
More than one	12/66 (18)	14/66 (21)	
Blood product transfusion	49/66 (74)	13/66 (20)	< 0.0001
Mechanical ventilation	57/66 (86)	53/66 (80)	0.484
Vasoactive drugs	41/66 (62)	25/66 (38)	0.009
Dialysis	23/66 (35)	2/66 (3)	< 0.0001
Type of dialysis†			
Hemodialysis	13/66 (20)	1/66 (2)	
Peritoneal dialysis	8/66 (12)	1/66 (2)	
Both	2/66 (3)	0/2	
Cardiopulmonary resuscitation	-	0/66	

HAART - highly active antiretroviral therapy.

* McNemar test (for paired samples) or when indicated ^†^, Fisher’s exact test or Chi-square test (for independent samples), both two-tailed tests;

‡† the sum was greater than 100% because some patients were included in more than one category.

In the initial evaluation by the palliative care team, 86% (57/66) of the terminally ill patients with records available were on mechanical ventilation, 62% (41/66) were using vasoactive drugs, and 35% (23/66) were under renal replacement therapy. After the evaluation by the medical team, the number of patients on mechanical ventilation decreased to 80% (53/66; p = 0.484), the use of vasoactive drugs decreased to 38% (25/66; p = 0.009), and the use of replacement renal therapy decreased to 3% (2/66, p < 0.0001). None of the 66 evaluated patients were subjected to cardiopulmonary resuscitation.

The analysis of the available hospital records indicated that meetings between the palliative care team or treating physicians and the family were held in 48% (52/109) of the cases. Among the terminally ill patients, meetings were conducted in 43% (32/74) of the cases. Regarding the ICU discharge of patients with available records, 28% (30/109) of the patients were transferred to the ward. Among the terminally ill patients, 23% (17/74) were discharged from the ICU (Table 4).

**Table 4:** Meetings with the family and patient discharge from the intensive care unit considering the total number of patients (N = 109) and terminally ill patients (N = 74)

	Total	Terminally ill patients
	N (%)	N (%)
Meetings with the family		
Yes	52 (48)	32 (43)
No	50 (46)	37 (50)
Unknown*	7 (6)	5 (7)
Discharge from the ICU		
Yes	30 (28)	17 (23)
No	63 (58)	49 (66)
Unknown*	16 (15)	8 (11)
Total	109	74

ICU - intensive care unit.

* Hospital records not available.

## DISCUSSION

In this study, the requests for a palliative care consultation involved the evaluation of patients with severe illnesses and high mortality; however, these consultations were often requested late. Therefore, only 15% of the patients who died in the ICU were evaluated by the palliative care team. Among the patients evaluated, the reasons for ICU admission included respiratory failure in 46% of the cases, diseases related to the diagnosis of HIV/AIDS in 71% of the cases, active opportunistic infection (predominantly tuberculosis) in 82% of the cases, and a CD4 count lower than 100cells/mm^3^ in 70% of the cases. Moreover, only 19% of the patients adhered to HAART, whereas irregular treatment or dropout occurred in 59% of the cases. These results suggest that most of these patients may have a significant social vulnerability that worsens their condition. The mortality of patients with requests for consultations was high (88%), and notably, 19 patients (15% of total requests) died before the first evaluation. These results suggest a possible delay in the requests for palliative care consultations, and this delay may have limited the adoption of appropriate palliative measures. This finding may be explained by the difficulty of defining the prognosis in HIV/AIDS patients despite the high prevalence of factors associated with higher mortality. With respect to the CD4 count, we observed a low cell count in most patients, although the CD4 count is not an isolated predictor of poor prognosis in the short term.^(4,21)^ A parameter that has been used recently is the immunovirological status at admission.^(22)^

We separately analyzed the patients predicted with a terminal illness. In this group, mortality was even higher than in the total sample of patients evaluated by the palliative care team (99% versus 88%). Even for patients with a prognosis of a non-terminal illness and with indeterminate prognosis, the mortality rate was almost two-fold the overall mortality rate of ICU patients evaluated during the study period. These findings suggest that palliative care consultations were often requested for cases of terminal illnesses. These results suggest that other benefits for requesting the palliative care team, including management of symptoms and support for the family, may help integrate intensive and palliative care.^(8,9,23)^

After the evaluation of the patients by the palliative care team, there was a significant reduction in the use of blood products, antibiotics, prophylaxis for opportunistic infections, and HAART for the terminally ill patients. Regarding the use of artificial life support, the use of vasoactive drugs and hemodialysis significantly decreased; however, the use of invasive mechanical ventilation did not significantly change. None of the evaluated patients underwent cardiopulmonary resuscitation. Most deaths in the ICU are preceded by the decision to limit treatment in any way, such as not escalating current interventions, not performing future interventions, and suspending some or all of the interventions except for those essential for promoting comfort.^(17,23-25)^ Specifically, in terminally ill patients with HIV/AIDS, there is no evidence supporting the maintenance or discontinuation of HAART. The potential benefits of maintenance include protection against HIV-related encephalopathy, relief of constitutional symptoms associated with the high viral load, and psychological comfort derived from disease management. The risks include adverse effects, drug interactions with medications used to relieve symptoms, limited therapy due to the unavailability of intravenous medication, the possibility of low absorption, which can lead to suboptimal medication levels, and the development of resistance.^(21,22,26)^ We found a high prevalence of tuberculosis in our patients, and antituberculosis drugs were used in almost all patients after the diagnosis of terminal illness, probably to decrease the number of smear-positive samples and consequently disease transmission. Antituberculosis drugs in palliative care are generally used for the treatment of multidrug-resistant tuberculosis. In this context, the discontinuation of this medication is indicated only in exceptional cases.^(27)^ However, if we consider that critically ill patients in the final stages of life have poor oral intake and poor intestinal absorption together with the possible toxicity of and interactions between TB drugs, the benefit of the suspension is greater than that of the maintenance.

Notably, 23% of the terminally ill patients were discharged from the ICU, although 94% (16/17) of the patients died in the ward during hospitalization. These findings indicate the potential benefit of joint monitoring of the patients by the palliative care team to decrease the number of inappropriate interventions for patients in the final stage of life, to prevent these patients from suffering social isolation, which is usually associated with the ICU, and to better allocate public health resources.^(17,28)^

Another significant finding of this study was the prevalence of family meetings, which occurred in only 46% of cases involving terminally ill patients. The study design did not allow us to infer the reasons for this low percentage, although we hypothesized that this situation was due to the high proportion of social vulnerability among hospitalized patients, which led to increased institutionalization and fewer family visits. Regardless of the cause, we believe that this finding indicates a potential for improvement because end-of-life care decisions should ideally be shared with patients and/or families to avoid the risks of unilateral decisions that are not focused on the best interests of the patients.^(29,30)^ A potential for improvement in these cases is the creation of clinical bioethics committees, which can propose and endorse medical decisions for institutionalized patients unable to make decisions.

Our study has some limitations. First, we only used data from the FAICP and hospital records, and the analysis of some variables may have been hindered by missing or incorrect data. Second, the unavailability of records from the SMAS limited the sample size and the amount of data available; additionally, the sample size was not calculated, and the inclusion of patients in a seven-year period limited the sample size. Third, the study population included only patients with requests for palliative care consultations, which limited the establishment of comparisons with other ICU groups. Finally, our study was conducted in a single hospital with very specific characteristics regarding the care services provided, which limited the data interpretation and generalization.

## CONCLUSION

The evaluation of patients by the palliative care team was restricted to a small number of patients in the intensive care unit. The requests for a palliative care consultation were made by patients with severe illnesses and high mortality, and these consultations were often requested late. The number of potentially inappropriate interventions in the patients monitored by the palliative care team and identified as terminally ill was significantly lower, and a few of these patients were discharged from the intensive care unit. This study identified several potential improvements and possible benefits of the integration of palliative care and intensive care. However, other prospective and interventional studies are necessary to better elucidate this association.
